# Performance and safety of a novel, cable-free, patch-based, and AI-enhanced ECG monitoring system: a comparative study

**DOI:** 10.1093/ehjdh/ztaf059

**Published:** 2025-05-26

**Authors:** Owain Thomas, Rikard Linnér, Alain Dardashti

**Affiliations:** Anesthesiology and Intensive Care, Department of Clinical Sciences, Lund, Section II, Skånes Universitetssjukhus Lund 221 85, Sweden; Anesthesiology and Intensive Care, Department of Clinical Sciences, Lund, Section II, Skånes Universitetssjukhus Lund 221 85, Sweden; Thoracic Surgery, Department of Clinical Sciences, Lund, Section II, Skånes Universitetssjukhus Lund 221 85, Sweden

**Keywords:** ECG monitoring, ECG telemetry, ECG patch, Life-threatening arrhythmia alarms, Critical arrhythmia alarms, Alarm fatigue

## Abstract

**Aims:**

ECG monitoring is often required during critical phases of illness. To evaluate the role of modern technology and advanced analytical algorithms artificial intelligence compared with standard-of care, we undertook a prospective, head-to-head comparison of a novel, cable-free, patch-based, and AI-enhanced electrocardiography system (CardioSenseSystem) with standard of care (SOC) ECG monitoring. Patients who had undergone cardiac surgery at a large university hospital (Skåne University Hospital, Sweden) were simultaneously monitored by both systems, and alarms and monitoring interruptions were recorded.

**Methods and results:**

Forty-nine patients were recruited. The CardioSenseSystem system demonstrated significantly higher sensitivity, correctly detecting 364 critical red alarms vs. 12 for SOC (*P* < 0.0001), and lower rates of high priority false alarms (0.3% vs. 40%; *P* < 0.0001). Monitoring interruptions were markedly reduced (114 s/day vs. 584 s/day; *P* < 0.0001). Handling time per patient day was significantly shorter (256 s vs. 880 s). The CardioSenseSystem system also reduced alarm fatigue, with fewer disturbances per patient per hour (0.03 vs. 0.11; *P* < 0.0001).

**Conclusion:**

The CardioSenseSystem system delivered significant advantages over conventional ECG monitoring in post-cardiac surgery patients. Its high sensitivity, reduced false alarms, fewer monitoring interruptions, and decreased handling time suggest that it may enhance patient outcomes and clinical efficiency, warranting broader application in acute-care settings.

## Graphical Abstract

**Graphical Abstract:**
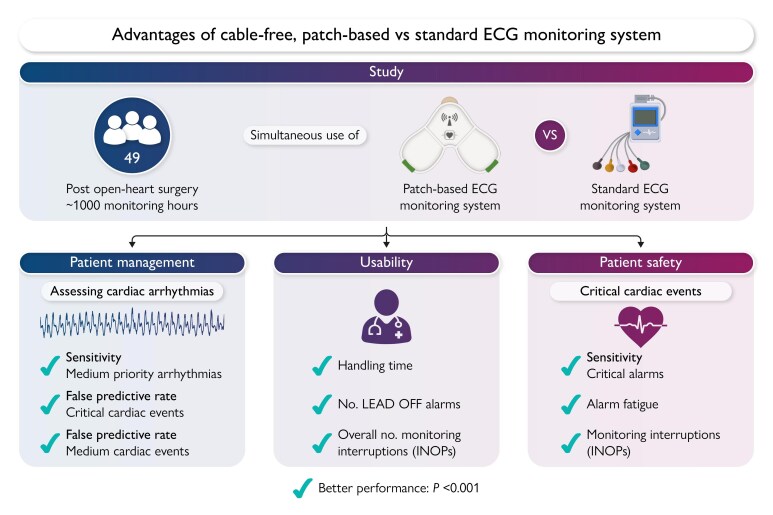

## Introduction

Continuous ECG monitoring has been a cornerstone in the management of patients at risk of cardiac arrhythmias for many years.^1^ But despite its well-documented benefits,^2–8^ traditional cable-based ECG systems, which have long been the standard of care (SOC), have several shortcomings. These fall into three categories: sensitivity, specificity, and reliability.

Sensitivity and specificity are often inversely related. If a monitoring system is set up to sensitively detect a cardiac event, it may also trigger numerous false events.^9^ On the other hand, SOC monitoring systems often fail to detect cardiac events—they lack sensitivity.^10^ The third category, reliability, refers to failure of monitoring because of some technical problem, for example, electrodes or cables becoming disconnected. Any interruption in monitoring is a potentially critical situation, as it leaves the patient unmonitored and hence at risk of an undetected cardiac event.

SOC monitoring systems can also degrade patient welfare and comfort. The physical constraints of ECG cables restrict patient movement and may make routine activities problematic, such as showering or visiting the toilet.

In this context, we evaluated the effectiveness of the ‘CardioSenseSystem’, a next-generation cable-free, wireless continuous ECG monitoring system. The CSS uses self-contained, independent ECG patches placed on the torso, each functioning as a standalone sensor that transmits data directly to access points, eliminating the need for cables. Our objective was to compare sensitivity and specificity with SOC, while minimizing monitoring interruptions and handling time in a high dependency setting for post-cardiac surgery patients. In addition, CSS incorporates advanced analytical AI algorithms, which we wanted to evaluate in this context.

## Methods

### Patients

Male or female patients at least 18 years old who were scheduled for cardiac surgery and in need of ECG monitoring in the Department of Thoracic Surgery, Skåne University Hospital, Lund, Sweden, between 18 May 2021 and 5 November 2021, were eligible for this study. Additional inclusion criteria were expected alarms in ECG monitoring during 24 h (that is, patients with known pre-operative arrhythmia, patients undergoing surgery due to unstable angina, or patients undergoing surgery for valve or aortic disease), and the ability to give voluntary, informed consent.

Patients were excluded if they had burns; known allergy or sensitivity to any of the CardioPatch components; infections in the area of electrode placement; brittle skin (e.g. after long-term cortisone treatment); open sternum; mechanical heart, extracorporeal membrane oxygenation; or an implanted defibrillator. Terminally ill patients were excluded, as were patients participating in another clinical investigation or whose participation could be detrimental to the patient or could risk obstructing the approved conduct of the investigation, at the discretion of the investigator.

### Ethical approval and patient consent

The study was approved by the Swedish Medical Product Agency (Study ID5.1-2018-18343) and the Swedish Ethical Review Authority, Lund (2019-04174). All patients received written and verbal information regarding the investigation. Before any investigation-related procedures, a consent form was signed and personally dated by both the patient and by the person who conducted the informed consent discussion. This clinical investigation was performed in compliance with Council Directive (MDD) 93/42/EEC, ISO 14 155:2011/14155:2020 and the ethical principles of the latest revision of the Declaration of Helsinki as adopted by the World Medical Association.

Although Novosense AB, the developer of the investigational device (CardioSenseSystem—CSS), provided technical support, the study was conducted under strict measures to ensure impartiality and scientific independence. The study was planned in collaboration with Novosense AB (Sponsor), Statcons (Biostatistics), and Devicia AB (Regulatory Compliance), with Skåne University Hospital responsible for and leading the study. The Swedish Medical Products Agency (Competent Authority) approved the study and provided detailed feedback on the design, methodology, and statistical considerations.

To ensure objective oversight and data integrity, the study was independently monitored by TFS HealthScience (GCP compliance), and data analysis was conducted by a third party, Key2Compliance AB. Following completion, the investigation and full clinical documentation were reviewed in detail by BSI (Notified Body), which confirmed not only the impartiality of the study but also the overall quality and integrity of the clinical data and documentation, supporting the CE marking of the CardioSenseSystem under MDR (certificate no. 766007).

### Investigational system

The continuous ECG monitoring system investigated in this study, ‘CardioSenseSystem’ (CSS), was developed by Novosense AB, Lund, Sweden (www.novosense.se), to support patient management and clinical decisions in patients admitted to hospital at risk of cardiac arrhythmias or myocardial ischaemia. At the heart of the CSS are three, single-use wireless sensors (CardioPatch). These are located on the patient to provide the derived (cable-free) equivalent of 6 conventional limb leads (I, II, III, aVR, aVL, and aVF), with Mason–Likar placement. A key innovation in this novel system is that each CardioPatch sensor effectively acts as an independent ECG monitoring system. Proprietary software leverages this redundancy to rapidly exclude less reliable signals, with the aim of improving sensitivity, specificity and reliability.

The CardioPatches are supplied as sets of three within a sealed tray and are pre-loaded with adhesive and hydrogel. Prior to packaging, each CardioPatch undergoes a cleaning procedure, which includes a wash with a water and surfactant solution (8–15%), followed by a final rinse with an alcohol solution. Once attached to the patient the CardioPatch sensors auto-calibrate within 1 min. The CardioPatch is designed to operate for 24 h (extended to 48 h in subsequent versions) and is then returned to the manufacturer for full refurbishment, making it a single-use device for end users.

**Figure 1:**
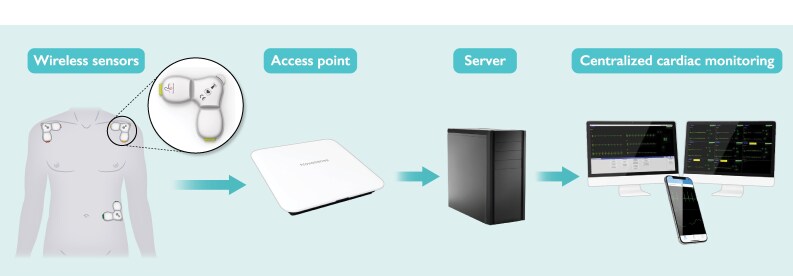
Components of the CardioSenseSystem. The CardioSenseSystem includes the following components, listed from left to right: the CardioPatch, three ECG patches, a wall-mounted access point, a server, and an information centre. The system supports presentation of alarms, real-time ECG monitoring, and retrospective analysis.

Each CardioPatch sends ECG data to a receiving station (Access Point), which can handle incoming data from multiple patients. These data are further processed by advanced proprietary AI algorithms on an intermediary server before being displayed on the ‘Novosense Information Centre’. This is the central user interface that allows ECG waveforms from up to 16 patients per station to be selected and monitored simultaneously; detects and generates alarms for 28 different events/arrhythmias according to user-defined parameters; and supports the analysis of multiple variables, including trends, alarms, waveforms, and ECG intervals (*Figure 1*).

The 1-min auto-calibration procedure involves measuring the electrical potential between the CardioPatches (corresponding to leads I and III) using a disposable thin cable, which is removed upon completion. This calibration phase captures full limb-lead ECG signals, which serve as a true reference for training a supervised AI model—that is, training the model with known outputs. The model learns to infer equivalent, cable-free limb-lead signals directly from the local ECG data collected by the three CardioPatches. As a result of this process, the derived and measured limb leads are typically indistinguishable during continuous monitoring.

The AI system responsible for alarm generation employs a two-stage supervised learning framework. In the first stage, patient-specific ECG morphology is modelled using time-aligned beats and outputs from a rule-based, multi-lead QRS detector. This supervised setup provides high-quality training data for the AI model. In the second stage, the model is used to enhance beat detection and classification—improving performance in the presence of noise and artefacts while increasing QRS classification accuracy.

QRS detection and beat classification are performed independently on each ECG signal, enhancing robustness through redundancy. This distributed detection strategy supports the system’s alarm logic, which relies on ensemble decision-making to achieve high sensitivity and specificity in real-time arrhythmia detection. Diagnostic accuracy is maintained even in the presence of motion artefacts, posture changes, or evolving cardiac conditions, resulting in a resilient ECG monitoring platform.

The alarm performance was verified prior to this study against several ECG databases, including the AHA Database, the MIT-BIH Arrhythmia Database, and the Creighton University Ventricular Tachyarrhythmia Database.

### Reference system (SOC)

The basis for the comparisons was the widely used (and SOC at Skåne University Hospital) Philips IntelliVue MX40, connected to IntelliVue Information Centre iX. This reference system uses five physical cables and ECG electrodes configured using the ‘EASI’ lead system, generating a calculated 12-lead ECG. The cables connect to a bedside receptacle from which the signal is led to a central unit for analysis and projection on monitors.

### Study design

This was a direct, head-to-head comparison of two continuous ECG monitoring systems in patients following cardiac surgery, the investigational and reference systems (above). Each patient in the study was simultaneously connected to the two systems. ECG waveforms from each patient were displayed at two separate central monitoring stations after passing through the respective central servers, with one monitor for each of the two ECG systems.

Throughout the study, any medical treatments or other devices required for the optimal care, safety and well-being of patients were supplied at the discretion of the study investigators as directed by hospital clinical protocols and routines.

### ECG alarm protocol

The physiological data in this study comprised 28 alarms (*Table 1*) divided into two categories: high priority red alarms (R1–5) and lower priority yellow alarms (Y1–23). Red alarms were triggered in response to critical cardiac events requiring immediate medical attention and included asystole, ventricular fibrillation, ventricular tachycardia, tachycardia, and bradycardia. As the reference device, SOC determined the values used in setting alarm sensitivity for each alarm. For example, extreme tachycardia was set at 170 beats per minute (b.p.m.) and tachycardia at 150 b.p.m. Importantly, all alarm settings were cross-checked at the beginning and end of each patient recording to ensure rigorous equivalence between the two monitoring systems.

**Table 1:** ECG system alarms for cardiac events in clinical study

Alarm event	Prefix	Description
Asystole	R1	Asystole (no QRS complexes detected for ≥ 4 s).
Ventricular fibrillation (VF)	R2	VF, rapid, erratic heartbeats originating from the ventricles. ECG displaying irregular activity without discernible pattern.
Ventricular tachycardia (VT)	R3	VT, rapid but regular heart rate originating from the ventricles. The alarm is triggered for ≥ 5 consecutive ventricular beats with a heart rate ≥ Threshold (100 b.p.m.).
Extreme sinus tachycardia	R4	Heart rate increased to potentially dangerous levels (> 170 b.p.m.).
Extreme sinus bradycardia	R5	Heart rate decreased to dangerously low levels (≤40 b.p.m.).
Multi ST segment changes	Y1	More than one lead has triggered an ST-alarm, see below.
ST elevation	Y2	ST elevation ≥ 0.2 mV. Detected on one lead.
ST depression	Y3	ST depression ≤ −0.2 mV. Detected on one lead.
QTc high	Y4	Prolonged QT interval corrected for heart rate. (QTc > 460 ms).
dQTc high	Y5	The delta QTc measures the change of the QTc interval (dQTc > 60 ms).
Non-sustained VT	Y6	Non-sustained ventricular tachycardia (VT ≤ 5 consecutive beats).
Ventricular rhythm	Y7	A run of ventricular beats (11 consecutive beats and < 100 b.p.m.).
Run PVCs	Y8	>Two consecutive ventricular beats but fewer beats than the Ventricular Rhythm’s alarm.
Pair PVCs	Y9	Two consecutive ventricular beats.
Pacer not capturing	Y10	No beat detected within 350 ms after pace pulse.
Pacer not pacing	Y11	No QRS and pace pulse for 1.75 times the average R-R interval.
Missed beat	Y12	No beat for 1.75 times average R-R interval for a heart rate below 120, or no beat for one second for a heart rate exceeding 120 (non-paced patient).
Pause	Y13	No new heartbeat is detected for 1.5–2.5 s. (2.5 s).
Supraventricular tachycardia (SVT)	Y14	Rapid heart rhythm originating above the ventricles.
*R-on-T PVCs	Y15	Ventricular beat occurring on the T wave of the preceding beat.
Ventricular bigeminy	Y16	A dominant rhythm of beats labelled as N^a^, V^b^, N, V, N.
Ventricular trigeminy	Y17	A dominant rhythm of beats labelled as N, N, V, N, N, V.
Frequent PVCs	Y18	The number of pre-mature ventricular complex (PVCs) during the last minute is greater than the PVCs/min threshold (10 PVCs/min).
Multiform PVCs	Y19	The occurrence of two differently shaped beats labelled as V within the last 60 beats and each occurring at least twice within the last 300 beats.
Sinus tachycardia	Y20	Heart rate exceeds the upper threshold (≥150 b.p.m.).
Sinus bradycardia	Y21	Heart rate is below the lower threshold (≤50 b.p.m.).
Atrial fibrillation (AFib)	Y22	Chaotic and irregular electrical impulses in the atria disrupt the heart’s normal rhythm, resulting in an uncoordinated and irregular heartbeat labelled as N.
Irregular heart rate	Y23	An irregular rhythm of beats labelled as N between R-R intervals (R-R interval changes > 12.5%).

^a^N beat: Normal QRS complex.

^b^V beat: Ventricular QRS complex.

The accuracy and classification of ECG events were reviewed and validated by a specialist clinician. The specialist had full access to patient medical records, as well as ECG data before and after each event, which was categorized as either TRUE or FALSE. This contextual information significantly improved the accuracy and certainty in categorizing individual ECG events. An ECG alarm was categorized as FALSE if the alarm triggering criteria did not match the true characteristics of the event with 100% accuracy. For example, a RUN PVCs event that triggered an alarm as Pair PVCs would be considered incorrect.

Due to the differing interfaces of the SOC and CSS systems, it was not feasible to blind the reviewer to which system was being evaluated. However, to minimize potential crossover bias, ECG events from the two systems were not assessed consecutively.

### Technical alarms

In addition to physiological alarms, each monitoring system triggered red and yellow technical alarms. Red, high priority/hard alarms indicated that the patient may no longer be monitored: For example, ‘ECG lead-off’ would be a high priority alarm. The lower priority alarms indicated operational issues, such as low battery (but still in operation).

### Handling time

In parallel with collecting data on the number and category of physiological and technical alarms, we also measured the handling time for each of the two monitoring systems. Parameters included the application of cables and electrodes (i.e. the initial set up time for each of the systems); any additional electrode/cable handling, such as following a lead-off or other disturbance; battery changes (not needed with CardioPatch, which is a self-contained single-use device); and any equipment cleaning and disinfection (estimated at 0.25 times/day). Again, this was not necessary with ‘CardioPatch’, which comes as a sealed, single-use device. Since only the time spent actually with the patient to deal with these technical matters was recorded, we also added a further 120 s per event as a standard ‘preparation time’ supplement to cover time taken to move to and from a patient, collection of consumables, and other logistical necessities.

### Statistical analysis

Initial sample size calculations indicated that 57 patients would deliver the necessary statistical power to compare the two ECG monitoring systems. We rounded this up to a target sample population of 60 patients. However, there were limited advance data available for this calculation, and therefore the study was set up with an interim analysis after 40 patients. The study was designed to continue after the inclusion of 40 patients but be terminated if statistically significant differences were found. A final analysis would then be conducted on all included patients. To compensate for advance interim analysis the confidence level was set at 97.5% to account for a Family Wise Error rate of 5% at both interim and final analyses. This adjustment was made to accommodate the possibility of terminating the investigation based on interim results.

In terms of the variables analysed in this study, our goal was to look for statistically meaningful differences across all key parameters. However, without prior knowledge of where differences would be found and to avoid the risk of introducing random significances, we employed the standard, robust analytical approach of first analysing an arbitrarily designated primary variable (monitoring interruptions) and then the secondary variables (sensitivity, specificity, false alarm rate, and handling time).

Thus, for the primary variable, differences between SOC and CSS were analysed using a two-sided Wilcoxon signed-rank test. A *P*-value of <0.025 was considered statistically significant. Outliers were defined as values >3 SD from the mean. These outliers were replaced with the mean interruption time.

In the analysis of the secondary variables, two statistical methods were used: the two-sided Wilcoxon signed-rank test and Fisher’s exact test (two-sided). As these analyses were performed only on the final results, a *P*-value of <0.05 was considered statistically significant.

## Results

A total of 49 patients (41 male and 8 female) with a median age of 64 (SD 12.1) years were included in the study. Median body-mass index was 26.7 (SD 3.59) kg/m^2^. The most common diagnosis necessitating surgery was stable angina pectoris (16 patients), followed by aortic stenosis (8), unstable angina pectoris (7), and aortic insufficiency (6). A full breakdown of admission diagnoses for all 49 patients is presented in *Table 2*. A total of 998.16 h of ECG monitoring data were collected.

**Table 2:** Underlying patient diagnoses

Diagnosis	Number of patients (*N* = 49)
Stable angina pectoris	16 (33%)
Aortic stenosis	8 (16%)
Unstable angina pectoris	7 (14%)
Aortic insufficiency	6 (12%)
Mitral insufficiency	4 (8%)
Aorta aneurysm in ascending aorta	2 (4%)
Aortic stenosis, stable angina pectoris	2 (4%)
Mitral insufficiency, mitral stenosis	1 (2%)
Myectomy	1 (2%)
Pulmonary insufficiency (tetralogy of Fallot)	1 (2%)
Myectomy right ventricle outflow obstruction	1 (2%)

### Sensitivity and specificity

**Figure 2:**
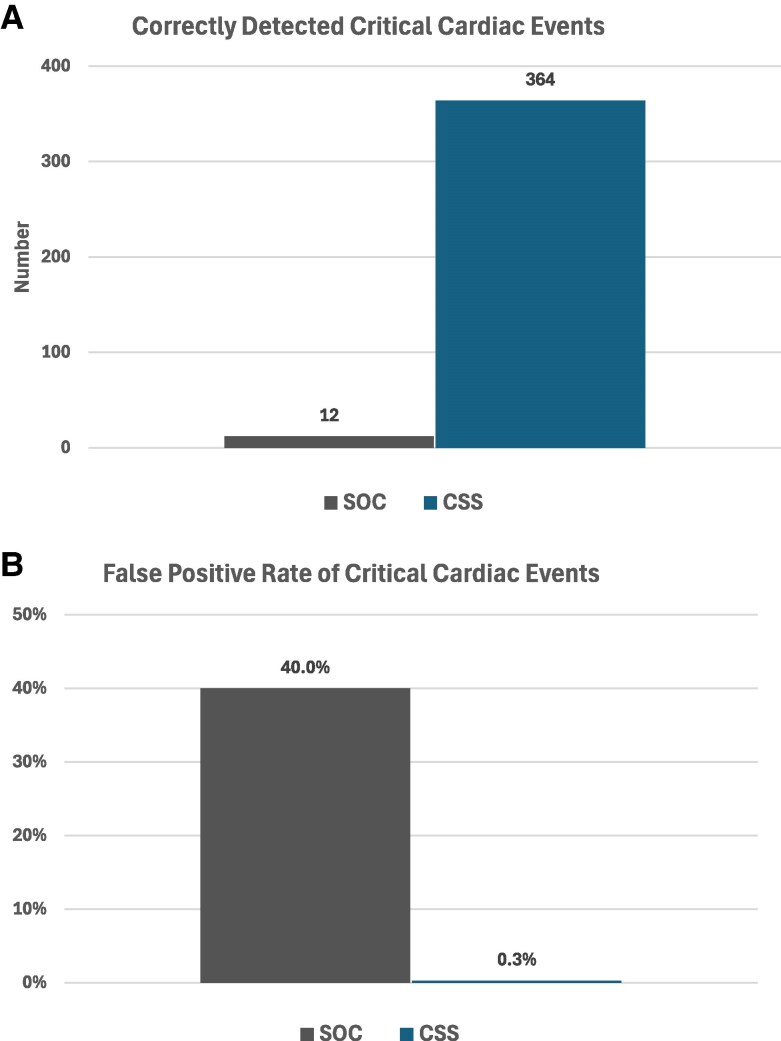
Critical cardiac events. (*A*) Number of correctly detected events; (*B*) false positive rate.

CSS correctly detected 364 high priority (red alarm) events, compared with 12 for SOC (*P* < 0.0001). In terms of false alarms, CSS falsely detected one high priority alarm (0.3%), while SOC falsely detected 8 (40%; *P* < 0.0001) (*Figure 2*). For lower priority (yellow) alarms, CSS correctly identified 1685 out of 1879 total alarms vs. 399 out of 581 for SOC (89.7% vs. 68.7%; (*P* < 0.0001). The total number of false alarms for lower priority (yellow) alarms was not significantly different between the two systems (194 vs. 182, respectively). A detailed breakdown of these data by alarm type and description is provided in *Table 3*.

**Table 3:** ECG alarm activation events, classified as true or false events by the physician

ECG alarm activation	SOC			CSS			*P*-value^a^
	Correct	False	Total	Correct	False	Total	
R1 Asystole	0	1	1	0	1	1	1.0000
R3 Ventricular tachycardia (VT)	3	6	9	3	0	3	0.1818
R4 Extreme Sinus Tachycardia	9	1	10	361	0	361	0.0270
Y6 Non-sustained VT	10	29	39	9	0	9	0.0001
Y7 Ventricular rhythm	0	2	2	0	0	0	1.0000
Y8 Run PVCs	3	7	10	0	0	0	1.0000
Y9 Pair PVCs	103	89	192	147	42	189	0.0001
Y10 Pacer not capturing	0	0	0	151	5	156	1.0000
Y11 Pacer not pacing	0	0	0	5	1	6	1.0000
Y12 Missed beat	16	32	48	850	119	969	0.0001
Y13 Pause	4	0	4	0	2	2	0.0667
Y14 Supraventricular tachycardia (SVT)	4	9	13	6	9	15	0.7055
Y18 Frequent PVCs	48	9	57	11	2	13	1.0000
Y20 Sinus tachycardia	81	1	82	378	1	379	0.3244
Y21 Sinus bradycardia	23	0	23	1	0	1	1.0000
Y22 Atrial fibrillation (AFib)	39	2	41	39	2	41	1.0000
Y23 Irregular heart rate	68	2	70	88	11	99	0.0756
Total red (R) alarms	**12**	**8**	**20**	**364**	**1**	**365**	**0**.**0001**
Total yellow (Y) alarms	**399**	**182**	**581**	**1685**	**194**	**1879**	**0**.**0001**
Total all ECG alarms	**411**	**190**	**601**	**2049**	**195**	**2244**	**0**.**0001**

^a^Fisher’s exact test (two-sided).

The bold numbers represent average values for red (R) critical alarms, yellow medium alarms, and the grand total of alarms. This should be clear from the text on the left. The bold numbers carry greater importance or weight than individual alarm type events.

Atrial fibrillation (AF) events often occur intermittently in patients, leading to a high volume of alarms. To address this, a timer functionality has been incorporated into both SOC and CSS systems to reduce the number of AF-related alarms. In this study, the AF alarm was programmed to deactivate if no AF was detected within 30 min. This feature was available and implemented in both monitoring systems. The *number* of AF alarms was similar for the two monitoring systems (*Table 3*; Y22), but the total *duration* of the alarm with CSS was five times longer than with SOC (113 vs. 21 h). This is of clinical importance as it allows insight into not only the occurrence but also the extent of AF over the previous 24 h.

### Monitoring interruptions

ECG monitoring interruptions are high priority events since they leave the patient vulnerable to the occurrence of an arrhythmia that goes undetected, and without potentially vital medical intervention. CSS lost 114 s per day due to interruption, compared with 584 s with SOC (*P* < 0.0001). A major contributor to monitoring interruptions are ‘lead-off’ events. Often these are transient alarms (lasting <30 s) triggered by patient movement. When such fleeting movement artefacts are removed from the analysis, high priority ‘lead-off’ technical alarms were almost eliminated by CSS (1 event) compared with SOC, which returned 36 (*Table 4*). CSS had 18 high priority ‘Cannot Analyse ECG’ compared with only 2 for SOC.

**Table 4:** Total number of technical alarms (INOPs), classified as high priority or transient alarm (<30 s)

Technical alarms (INOSOC) events	‘SOC’	‘CSS’	‘SOC’	‘CSS’
	High priority	High priority	Transient alarm	Transient alarm
Cannot analyse ECG	2	18	118	0
ECG leads off	36	1	11	6
Replace battery	10	0	0	0
Battery depleted	31	0	0	0
No signal	25	8	172	170
Total no. events	104	27	301	176

The SOC system, however, does not store the ‘Cannot analyse ECG’ alert in its alarm history. The ‘Cannot analyse ECG’ alert for SOC was manually entered by the investigator on sections that did not show any annotation of heart rhythm and had otherwise no other ‘inoperative’ (INOP) alarms activated. This often occurred before a ‘lead-off’ alarm. Furthermore, the SOC system performs some post-processing of the data, i.e. after the alarm generation. Thus there is a risk that the ‘Cannot analyse ECG’ event on the SOC system is underreported.

**Figure 3:**
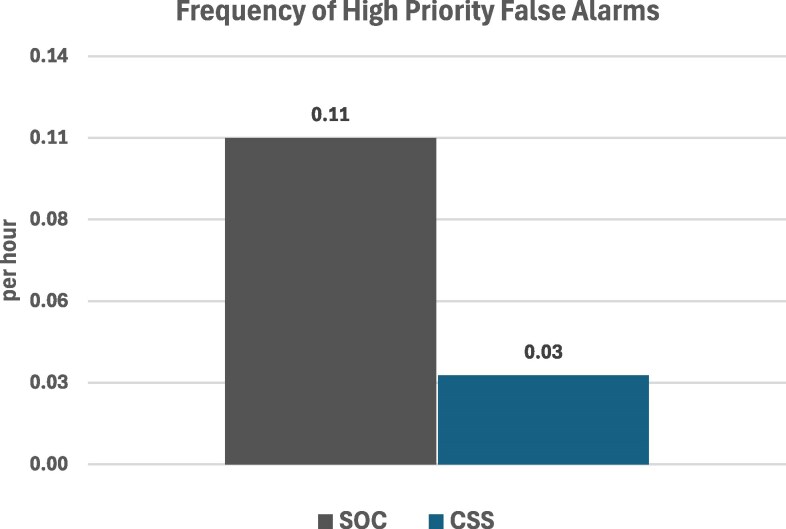
Frequency of false high priority alarms per patient.

These monitoring interruptions, together with false alarms for high priority cardiac events, contribute to the well-documented problem of ‘alarm fatigue’. CSS had significantly fewer of these ‘alarm fatigue’ events (28) than SOC (112) (*Tables 3* and *4*), amounting to a much lower rate of disturbances/hour (0.03 for ‘CSS’ vso 0.11 for ‘SOC’; *P* < 0.0001) (*Figure 3*).

### Handling time

The daily handling time (combined time for each activity plus preparation time where required) was 4 min 16 s for CSS and 14 min 40 s with SOC (*Table 5*).

**Table 5:** Handling time by activities performed

Activity	Mean handling time/activity (s)		Number of activities		Mean time per patient day (s)	
	SOC	CSS	SOC	CSS	SOC	CSS
Application of cables/electrodes	186	83	56	49	250	98
Extra handling of cables/electrodes	99	50	37	4	88	5
Battery change	142	0	31	0	106	0
Disinfecting of equipment (estimated to 0.25 times/day)	190	0	12	0	48	0
Preparation time	120	120	134	53	388	153
Total handling time (sec)					880	256

### Safety

No patients experienced skin irritation, infection, or other adverse skin reactions during the study period.

## Discussion

Continuous ECG monitoring has been a cornerstone in the management of patients at risk of cardiac arrhythmias for more than 60 years. When first introduced onto specialist Coronary Care Units (CCU) in the 1960s, it played a key role in markedly decreasing mortality following acute myocardial infarction.^11–15^ Today, continuous ECG monitoring has extended well beyond the CCU and is central to patient management in the acute and emergency hospital settings, where rapid detection of abnormal cardiac activity is critical to improved patient outcomes.^2–8^

However, false alarms, often described as ‘non-actionable alarms’, have been repeatedly identified as a driver of ‘alarm fatigue’. This is a serious situation in which clinical staffs are desensitized to ECG warning alarms, resulting in large numbers of entirely preventable hospital deaths^16–18^ On the other hand, lack of sensitivity leads to failure to detect cardiac events. In one observational study, only just over a half of all cardiac arrests in almost 9000 patients were accurately detected.^10^

Technical alarms, for example those resulting from cables or alarms becoming disconnected, also add to alarm fatigue. In the ‘UCSF Alarm Study’, which included 461 intensive care unit (ICU) patients, there were more than 2.5 million alarms, with just over 30% being technical alarms, defined as ‘lead-off’, artefact, or other disconnection events.^19^ High numbers of false alarms also affect patients, by disturbing their rest, creating unease, and reducing confidence in treatment.

The emergence of wireless cardiac monitoring systems is a relatively new development in the healthcare sector, made possible by advances in software and in hardware across multiple areas of technology. There are currently few wireless systems available, most resembling traditional Holter monitors for outpatient ECG recording. The main difference between the CSS and Holter ECG patches, such as PSMAcpc, AT-Patch, SmartCardia, and Zio AT, is their intended use. The CSS is designed for continuous in-hospital ECG monitoring, primarily for critically ill patients at risk of rapid health deterioration. In contrast, patch-based ECG devices are typically used in home settings for identifying and diagnosing rare cardiac events. Consequently, the requirements for these systems differ significantly: the CSS must react quickly for critical care, while Holter patches store data for later analysis.

Another difference is that the CSS provides derived limb leads, which are familiar to specialists, while Holter patches use non-standard ECG recordings. Additionally, the CSS utilizes three independent sensors, which is likely a key factor in its superior performance compared with standard-of-care devices in the key endpoints of this study.

Early studies comparing patch-based ECG monitors to traditional telemetry systems in non-critical hospitalized patients have been rather disappointing.^20^

Recently, a small feasibility study compared a single-patch, single-lead wireless system with conventional ECG monitoring in patients following cardiac surgery. The findings suggested that both systems were broadly similar in sensitivity, with the wireless monitor triggering fewer false alarms. However, this study included only 190 cardiac events, and used data event storage procedures typically adapted for Holter ECG recording.^21^

To the best of our knowledge, our study is the first to compare a patch-based ECG monitoring system with conventional standard-of-care methods in an acute-care hospital setting. Unlike previous studies, this long-term investigation was designed for real-time alarm functionality, as required for in-hospital ECG monitoring. It also included continuous clinical oversight via a central monitoring station, using retrospective patient data to assist in patient management, treatment decisions, and the timely provision of medical interventions.

### Explaining accuracy and reliability

The findings of this study are notable in two key areas of continuous ECG monitoring in acute-care patients: (i) the detection of real events/arrhythmias and (ii) minimizing the burden of false, high priority alarms (false physiological alarms and technical alarms). The observed difference between the performance of CSS compared with SOC in key clinical areas may be attributed to the signal redundancy designed into CardioPatch. Each CardioPatch is independently grounded (Right Leg Drive, RLD), amplifying common mode signals and phase shifting these to eliminate signal-disrupting interference, such as from the mains electricity supply. As a result, if one CardioPatch becomes disconnected, or is adversely affected by motion artefacts or other interference, the other two patches can remain unaffected.

This is in marked contrast to conventional, cable-based systems where the disruption of one cable/electrode can have a severe impact on the overall ECG recording, particularly if that cable is RLD, in which case the system can no longer monitor ECG at all. With CardioPatch if one, or even two, sensors are partially or fully disconnected the system will only generate a lower priority ‘one lead-off’ alarm, during which the patient will continue to be fully monitored.

All technical alarms documented in this study were classified as ‘INOPs’ (inoperable), meaning that the patient is no longer being monitored. Lower priority alarms, such as ‘noise’, or ‘one lead-off’ were not documented, as SOC did not store these events in the history log. We defined lower priority technical alarms as those that last less than 30 s. This is compatible with real-world practice where brief alarms, usually resulting from movement and other interference artefacts, will automatically reset.

### Handling time matters

While handling time may not carry the same clinical consequences as sensitivity and the ‘alarm fatigue’ generating issues of specificity and technical alarms, it is of particular importance in an era of constrained healthcare resources. In our study, the reduction in nursing time, when applied to our entire ward (10 000 patient days per year), equates to approximately one full-time person equivalent per year.

Notably, each patient set of CardioPatch is single-use and enclosed in a sealed and cleaned package, which ensures consistent hygiene with no need for onsite disinfection. This, together with the significantly decreased time associated with maintaining ECG cables/electrodes and battery changes, contributes to the reduced handling time with CSS compared with SOC. Battery changes are not needed with CSS, as it a single-use device lasting 24 h (extended to 48 h in subsequent versions).

The CSS has received CE marking under MDR but is not yet available on the market. The company has indicated that it will be sold at a cost-neutral price compared with competitors, which would suggest a potential cost benefit due to the reduced handling time associated with CSS.

### Considerations

Our study was limited to patients who had undergone open-heart surgery in a cardiac unit at a single centre. While this focus may limit the generalizability of the results to other patient populations, it is important to note that this group is particularly well-suited for ECG monitoring. Post-operative cardiac surgery patients have increased risk for clinically significant arrhythmias—such as atrial fibrillation, bradyarrhythmias, and ventricular tachyarrhythmias—especially during the early recovery phase. Continuous ECG monitoring in this context is strongly supported by current clinical guidelines^22^ (Class I, Level of Evidence B), underscoring the relevance and appropriateness of selecting this cohort for initial evaluation of novel monitoring technologies. Furthermore, as these patients often recover quickly post-surgery and early mobilization is a key component of rehabilitation, the monitoring technology must meet high standards for reliability, flexibility, and patient comfort. The need for uninterrupted signal acquisition during movement places considerable demands on conventional systems, often leading to artefacts, alarm fatigue, or interruptions in monitoring. Although we have no reason to expect that the CSS would perform differently in other care environments, we fully agree that broader validation is essential. The current system has been validated for single-use with up to 24 h of continuous monitoring, during which no decline in performance or safety was observed. The system should be validated for extended periods—48 h or longer—with a focus on performance stability, skin tolerance, and signal quality throughout prolonged use. Future multi-centre studies across a range of clinical settings—including general wards, ICUs and emergency departments—are encouraged to evaluate system performance in more diverse patient populations and operational contexts. Together, these future investigations will help ensure that technologies like CSS remain robust, safe, and clinically effective across real-world conditions and extended use cases.

Skin irritation was identified as a potential adverse event in the Clinical Investigation Plan and was therefore included as one of the monitored safety variables during the study. No patients experienced any signs of skin irritation. Given the very low rate of ‘LEAD-OFF’ events observed during the study, one might expect the sensors to adhere too strongly, potentially causing discomfort during removal. However, removal of the CardioPatches at the end of the study was surprisingly easy and well-tolerated. This was likely due to the two-stage removal process: first, the silicone sensor was detached, leaving the adhesive on the patient’s skin; then, the adhesive—equipped with a pull tab—was removed by peeling it off at a 180° angle.

We did not make any direct comparative assessments of the two monitoring systems on patient comfort or well-being. We believe this would be a valuable area for future investigation. Informal feedback from participants suggested high levels of comfort with the CardioPatch, consistent with its cable-free design. Unlike traditional ECG monitors—where cables can become tangled or dislodged and telemetry boxes must be worn or carried—the CardioPatch can be applied and largely forgotten, providing a more seamless patient experience. While direct measures of patient comfort were not collected, several indirect indicators support the notion of improved patient experience with CSS. The low rate of false and technical alarms, particularly among high priority alerts, suggests a lower potential for alarm-related anxiety or disturbance compared with standard care. Additionally, the freedom from cables likely supports greater patient mobility and the waterproof design enables patients to shower without the restrictions typically associated with traditional ECG systems. Together, these features may contribute to a more comfortable and less intrusive monitoring experience, warranting further exploration in future studies focused on patient-centred outcomes. This positive reception is further reflected in the exceptionally high participation rate: due to the broad inclusion criteria and the non-invasive nature of CSS, the threshold for participation was low, and very few eligible patients declined consent.

## Conclusion

In this clinical trial, which accumulated almost 1000 h (998.16) of ECG monitoring data from 49 patients, CSS was found to be significantly better than SOC in terms of the key study parameters of sensitivity (accurately detecting cardiac events), specificity (minimizing the number of high priority false alarms), and reliability (limiting the number and duration of monitoring interruptions). Importantly, CSS was also associated with marked reductions in staff handling time. In our opinion, the advantages were achieved by the combination of novel hardware (cable-free, wireless patches), with inherent high redundancy from the three parallel independent channels linked to the advanced analytical AI algorithms of the CSS-system.

## Data Availability

The data underlying this article will be shared on reasonable request to the corresponding author.
